# Assessment of the obesogenic environment in primary schools: a multi-site case study in Jakarta

**DOI:** 10.1186/s40795-022-00513-y

**Published:** 2022-03-04

**Authors:** Levina Chandra Khoe, Indah Suci Widyahening, Syougie Ali, Helda Khusun

**Affiliations:** 1 Department of Community Medicine, Faculty of Medicine Universitas Indonesia, Pegangsaan Timur 16, Jakarta, 10430 Indonesia; 2 Southeast Asian Ministers of Education Organization – Regional Centre for Food and Nutrition (SEAMEO-RECFON)/Pusat Kajian Gizi Regional (PKGR), Universitas Indonesia, Jakarta, Indonesia

**Keywords:** Obesity, School, Nutrition environment, Children

## Abstract

**Background:**

Childhood overweight and obesity have increasingly been recognized as a significant global public health crisis, including in Asia. This study aimed to assess the obesogenic environment in primary schools in Jakarta, Indonesia.

**Methods:**

A qualitative, multi-site, case study design was used to capture different elements of the school environment and policies related to obesity, with a focus on nutrition and physical activity. An adaptation of the Primary School Environmental Assessment tool was used. Six primary schools in Jakarta were purposively selected based on their location, socioeconomic status, and type (public or private). In addition to direct observation at each school, interviews were conducted with the principal, physical education teacher, canteen staff, street food vendors, and students.

**Results:**

Among the six schools, two were private and four were public. The most popular foods consumed by students were unhealthy, such as deep-fried foods and sugar-sweetened beverages. Students had easy and constant access to unhealthy foods, whereas only limited variation of healthy foods were available in the school canteen. Some schools also allowed the student to have access to street food vendors. School policies related to healthy eating and physical activities had been implemented, mainly in the form of teaching these topics as part of the school curriculum. However, promotion of healthy eating and physical activities by the schools was still limited.

**Conclusions:**

This study showed the usefulness of the Primary School Environmental Assessment tool in identifying obesogenic factors in urban area of Indonesia. Effective implementation of guidelines to foster good nutritional practices and healthy lifestyles at school should be prioritized to improve the health and nutritional status of the students.

## Background

Childhood overweight and obesity have increasingly been recognized as a significant global public health problem [1]. Asian countries like Indonesia are currently challenged by the double burden of an increasing prevalence of overweight and obesity and the persistence of under-nutrition. The rate of childhood stunting remains high, around 30%, while the prevalence of overweight children age 6–12 years has increased from 5.1% in 1993 to 15.6% in 2014, particularly in urban areas [2]. In most cases, childhood obesity may continue into adulthood and lead to chronic illnesses and premature mortality [3, 4].

Various factors contribute to childhood obesity, such as unhealthy diet; lack of physical activity, parental education, nutrition education at school; family stress; inadequate sleep; and increased television viewing [5–9]. The risk factors for obesity are combination of lifestyle and environmental elements that influence the individual’s food consumption habits. In a child’s life, both home and school play a vital role in creating a positive lifestyle and supportive environment for healthy eating.

Children spend a large portion of their day in school, where they also spend their time eating, drinking, and interacting with teachers and peers. In this environment, children may be exposed to obesity-related factors, such as meals at the school canteen, food vendors around schools, peer-influenced food choices, and elements in the school climate that may be associated with a poor level of physical activity as well as behavior problems [10]. However, although the school environment can present significant risk factors for childhood obesity, it can also provide a good opportunity for public health intervention [11].

An audit tool to assess the obesogenic food environment in primary schools, named the Primary School Environmental Assessment (PSEA), has been developed by Auckland University New Zealand [12] and was further adapted for use in Brunei Darussalam [13]. Our study aimed to explore the usefulness of the PSEA tool to assess obesogenic factors in the school environment in an urban area of Jakarta, Indonesia. The availability of such assessment tools would be helpful to develop school-based interventions and support decisions by policy-makers to prevent childhood overweight and obesity.

## Methods

### Study design

A multiple case study design was chosen in this study as it is considered as practical approach to understand a phenomenon comprehensively in its real-life context [14]. This study was conducted in Jakarta, in April to September 2019. Children in Indonesia typically start primary school at age 6 and the duration of the primary education is six years. Stake et al. recommends at least four cases to be explored in a multiple-case study design [15]. Six schools (two private and four public schools) were selected based on the recommendation of the local education office to represent different geographic areas and the socioeconomic status (SES) level of the area where the school is located (moderate, high, and very high SES based on the classification by Central Bureau of Statistics Indonesia).

### PSEA tool validation

The modified version of PSEA tool validated in Brunei Darussalam was chosen because the setting was considered culturally and demographically more similar to Indonesia compare to New Zealand. The tool contains eight sections, namely: school demographics; internal canteen service; external food service and outside food vendors; school food/nutrition policy; nutrition environment; school physical activity policy; physical activity environment; and external physical activity environment. It is a mixed-method tool, containing 5–16 close-and open-ended questions in each section. The close-ended questions have various options, such as yes/no, very much agree–very much disagree, very high–very low, and highly effective–not effective while the open-ended questions were asked when further information regarding the items was required or to solicit general comments on a condition [13].

The Brunei PSEA was first translated from English to Indonesia, and then translated back to English by two independent medical translators. The initial draft of Indonesian PSEA was then reviewed by a group of experts, including nutritionists, a sports medicine physician, a community medicine physician, teachers/education experts, and government authorities that were related to primary education, both from the central and local governments. Some modifications were made through the review process to further suit the Indonesian setting, such as adjusting the list of foods to those commonly found in Indonesian schools and deleting questions about vending machine.

### Data collection

Originally the PSEA questionnaire developed by Auckland University was made to be distributed to schools through mail and filled by the appropriate school administrators on their own. We choose to follow the data collection method conducted in Brunei which involved observation and direct questioning of the informants which enable us to obtain a more complete description of each component. In each school, the researchers conducted guided interviews using the PSEA tool with various informants: school headmasters or their deputies (PSEA Sects. 1, 3–5, 6), physical education (PE) teachers (Sects. 6 and 7), 1–2 students (Sect. 3), 1–2 school canteen staff (Sect. 2), and 1–2 street vendors around the schools (Sect. 3). The informants were selected purposively based on its role as policy makers, executor (in this case those who provide meals in schools), and users (i.e., students). The students involved in this study were chosen by their teachers, represented by those in the higher level of elementary school (level 4, 5, or 6) since they were more open to discussion. Interviewers were a school of public health graduate and a medical doctor. The close-ended questions in the PSEA tool were asked first followed by the open-ended questions to obtain more specific information. The responses to each question were recorded both in writing and in audio recordings. Interviews with the headmasters and teachers were conducted in their offices, while interviews with the students and school canteen staff were conducted in the canteen. The interviews with the street vendors were conducted in their respective working areas. The researchers also observed the physical condition of the schools and its surroundings (Sects. 2, 3, and 8) and asked for the price of the foods sold in the school canteens and by the street vendors. Before data collection, the researchers visited each school and explained the study objectives to the school principal. Permit letters from the education authorities and ethics committee were sent to the schools. Headmasters who agreed to participate in the study were then scheduled for an interview. The length of the interview was approximately 20–40 min per person, while the whole process of the interview and observation of each school was completed in one day. As mandatory by the ethical committee, we explained the aim of the study, the question components, and the importance of study to the respondents before the interview.

### Data analysis

We choose to organize the case study according to a descriptive framework as the general analytic strategy [16]. Individual interviews with the informants were utilized to obtain data about each school. Results from the interviews collected as written and audio records were combined with data from observations of the school environment and entered into an Excel spreadsheet according to the sections of the PSEA for each respective school. One of the authors (LCK) transcribed the audio recordings in consultations with the interviewers. The interview results of the close-ended questions are presented descriptively in summary tables (Table 1–3). Data from the open-ended questions were combined with data collected as written records, audio recordings, and photographs, and are presented as narratives and direct quotes accompanying the tables to further describe the findings. The main unit of analysis or the “case” in this study is the school, where its level of obesogenic environment was assessed using PSEA tool. Each school was treated as a single case study to provide an in-depth understanding of the phenomenon in each school. To synthesize data from the six schools, a pattern matching technique was used to compare the similarity and differences of each PSEA component across the six schools. Comparison was made between public and private schools. Thematic analysis was conducted by ISW and LCK, medical doctors who are trained in qualitative study methods and were involved in the validation of the PSEA tools and data collection. All of the analyses were performed manually based on the spreadsheets. Written records or transcripts of audio records were not returned to the respondents for further comments. Nevertheless, a workshop attended by the school’s principals, as well as the representatives of the local education and health offices were conducted at the end of the study to gain further clarification and feedback from the participants on the study results.

### Participants and Public Involvement

Participants and public were involved during the conduct of the study through the involvement of teachers/education experts, school’s representatives and government authorities that were related to primary education in the questionnaire adaptation process as well as the dissemination plan of the study result.

## Results

### School characteristics

All six schools contacted agreed to participate in the study. Two schools were located in an area with very high socioeconomic status, two were at the high level, and the other two were at the moderate level. There are no school located in the low socioeconomic area in Jakarta. The number of students ranged from 111 to 541 per school. In general, the schools had no or only one teacher trained in nutrition and health education, usually the PE teacher or the teacher in charge at the school health post. Both of the schools located in the moderate socioeconomic status (SES) have one teacher trained in nutrition and health education, however only one of the two schools located in the high and very high SES have such teacher. Two of the four public schools observed in this study shared buildings, sports fields and canteens with other schools. Another school shared the building/area with an orphanage. **Table ****1** describes the characteristics of the schools.

**Table 1:** Characteristics of the schools

	**School 1**	**School 2**	**School 3**	**School 4**	**School 5**	**School 6**
Public/private	Public	Public	Public	Public	Private	Private
Socioeconomic status	Moderate	Moderate	High	High	Very high	Very high
Total number of students	541	192	181	246	111	191
Male	282	105	104	117	54	98
Female	259	87	77	129	57	93
Number of teachers receiving nutrition training	1	1	0	1	0	1
Number of teachers receiving health training	1	1	0	1	0	1

### Food access and availability

**Table ****2** presents the food access and availability of each school based on components 2 and 3 of the PSEA tool. All schools have a school canteen within their premises, which is open in accordance with the school operation time. No healthy canteen guidelines existed in any of the schools. All of the canteens served a breakfast menu, and the most popular food choices were rice-based meals (e.g., fried rice, coconut rice, turmeric rice), fried/instant noodles/vermicelli, sweet breads/buns, spaghetti, and various deep-fried snacks. One public school canteen had vegetable soup on their menu. Sugar-sweetened beverages and instant (sachet) drinks were the most favorite drinks among the students in all schools.

**Table 2:** Food access and availability in primary schools based on the Primary School Environmental Assessment

	**School 1**	**School 2**	**School 3**	**School 4**	**School 5**	**School 6**
***Internal canteen service***						
** School canteen**	Yes	Yes	Yes	Yes	Yes	Yes
** Operational hours**	06.00–13.00	06.00–13.00	06.00–12.00	06.00–12.30	08.00–15.00	09.30–11.00
** Canteen operator**	Parents	Parents	School principal	Community	School staff	School staff
** Selling healthy food**	Sometimes	Yes	Yes	Sometimes	Sometimes	Never
** Promotion of healthy meals**	Daily	Daily	Occasionally	Never	Never	Never
** Canteen health inspection**	Yes	Yes	Yes	Yes	Yes	Yes
***External food service and outside food vendors***						
**External catering**	No	No	Yes	No	Yes	Yes
**Source of catering**	N/A	N/A	Committee of student’s parents	N/A	Parents	Ex-teacher
**Street vendors**	Yes	Yes	Yes	No	Yes	No

Almost all canteens had fruits or vegetables on their menu. However, the canteen staff in all schools remarked that fruits or vegetables were not popular among the students. One canteen in a private school never offered healthy meal options to the students because of the thought that no one is going to buy them. When the canteen staff was asked about healthy diet promotion in the menu, two canteens of the four public schools mentioned that they provided a healthy meal as a special menu of the day that is promoted occasionally. None of the two private school’s canteens ever promoted a healthy diet. The two schools in moderate SES area admitted promoting healthy meal in daily basis, while those in higher SES level only occasionally or never had promotion.

“There are vegetables every day, such as bean sprouts, mustard greens and tofu, and sometimes spinach. The children’s favorite meal is crispy chicken”

Canteen staff (school 5).

The canteen staff perceived healthy food as those without high content of sugar and salt, but still preferred by children. This would be difficult as children love sugary and savory food. We found that sugar-sweetened beverages and fried foods were highly popular.

“What is taken into consideration is that the snacks sold in the canteen, the food and drink itself, must be healthy, not high in sugar and salt, children like it, but should take into account the spices and the containers used.”

Canteen staff (school 6).

The food prices in this study are reported in United States (US) dollars and cents. Rice-based meals usually cost less than one dollar, whereas the cost for snacks (e.g., chips, crackers, variety of deep-fried snacks, sweets, and chocolate) was even lower (< 15 cents). Fruit costs approximately 7 cents, and fruit juices (with added sugar) costs higher (approximately 70 cents). The bottled (600 mL) mineral water costs 15 cents, whereas the costs for sugar-sweetened beverages and instant drinks were slightly higher, approximately 20 cents.

School canteens were regularly inspected by the local public health centers (Puskesmas). Some (2/6) were also inspected by the Indonesian Food and Drug Agency. Inspections also included the street food vendors in the surrounding area. All the canteen staff considered that routine inspection by the Puskesmas was sufficient to maintain the hygiene and quality of the canteen. None of the inspections took notice of the food variation and nutrition quality, as their main concern was to examine food hygiene and cleanliness. Catering services from external sources were also optional in some schools (3/6), especially in the private schools. These services were usually organized by parents or a former teacher. This external food source was provided at an additional charge to the students. The catering service provided a complete lunch box consists of rice, meat/chicken, vegetables, fruit, and dessert (e.g., ice cream/probiotic drinks).

Almost all schools had easy access to street vendors. Only one school did not have access to street vendors because it is located in a narrow alley, which limited the vendors’ access to the school environment. Most schools (5/6) did not have strict regulation that forbade their students to buy food from street vendors, but one private school banned their students’ access to street vendors.

“We do not know if the food outside the school is healthy or not; we never ask. We only know about the food inside the fence. "

Headmaster (school 1).

Deep-fried foods, ice cream, and sugar-sweetened beverages were the most common foods offered by the street vendors. The price for these foods was even lower than the foods sold in the school canteen. Because street vendors usually sell food in carts or a bicycle, they can easily come and go and thus are able to avoid inspection from authorities.

### School policies and environment in healthy eating

School policies and environment in healthy eating is presented in **Table ****3** based on components 4 to 8 of the PSEA tool. All school headmasters claimed to have a written policy that actively promotes healthy eating to students, which mainly integrated topics about healthy eating within the national school curriculum, such as in a natural science lesson. Two of the public schools and one of the private schools had additional program, such as having a weekly fruit day or weekly healthy breakfast and offering eating breakfast together with all students and teachers. Some schools (3/6) also claimed to regulate the type of foods that should be available in the canteen or in the student’s lunch box and provided information on healthy food and eating. Teachers delivered the messages through announcement in the class. Nevertheless, almost all schools admitted that they do not strictly monitor the implementation of these regulations.

**Table 3:** School policy and environment on nutrition and physical activities

	**School 1**	**School 2**	**School 3**	**School 4**	**School 5**	**School 6**
***Policy on food/nutrition and nutritional environment***						
** Written policy**	Yes	Yes	Yes	Yes	Yes	Yes
** Effectiveness of policy**	Quite effective	Quite effective	Not effective	Very effective	Quite effective	Quite effective
** Promotion on healthy eating**	High priority	High priority	Very high	High priority	Low priority	Low priority
** Teachers’ support**	Very good	Good	Good	Good	Good	Good
** Parental fort**	Very high	High	Very high	Very high	Moderate	High
** Sponsorship by industries**	None	None	None	None	None	None
** Written policy**	Yes	No	No	No	Yes	No
** Effectiveness of policy**	Effective	Effective	Effective	Very effective	Effective	Quite effective
** Duration of physical education class**^a^	2-h lesson per week for 1^st^–3^rd^ grade, 4-h lesson per week for 4^th^–6^th^ grade	4-h lesson per week	4-h lesson per week	2-h lesson per week for 1^st^–3^rd^ grade, 4-h lesson per week for 4^th^–6^th^ grade	2-h lesson per week	2-h lesson per week
***Physical activity environment (internal and external)***						
** Within school**	Adequate	Adequate	Adequate	Adequate	Adequate	Adequate
** Outside school**	Inadequate	Inadequate	Inadequate	Inadequate	Inadequate	Inadequate
** Support from teachers**	High	High	High	High	High	Moderate

^a^1-h lesson ≈ 35 min

The headmasters and PE teachers used the number of students who brought lunch box from home as a parameter to measure the effectiveness of the policy on nutrition, hence five schools claimed that the policy had been effective. One public school headmaster admitted that the regulations were ineffective because the canteen is typically fully occupied with parents who wait for their children; thus the children get food from the street vendors instead. However, he stated that the schools already planned to implement a new strategy the following school year.

"Starting next school year, the children are required to bring food from home, so their food is clean, they learn to be calm, and they wouldn't want to buy food from the street vendors as often."

Headmaster (school 3).

Headmaster of the four public-schools located in moderate and high SES area claimed that they placed a high or very high priority on the promotion of healthy eating. These priorities were shown by the presence of healthy diet posters around school and the implementation of healthy eating practices at certain events. Two other schools also provided health education in collaboration with the local public health center. Nevertheless, both private schools in the very high SES area assumed healthy eating was a low priority.

All headmasters and PE teachers considered teachers to be a good role model in healthy eating for students. Teachers usually brought their own lunchbox, chose healthier food from the canteen menu, promoted a healthy lunchbox to students, and reminded students to not buy food from the street vendors. They were also in agreement that parents are important factors and very supportive toward promoting healthy eating, especially for motivating students to bring lunch from home.

### Physical activities policy and environment

Physical activity was promoted through PE as a compulsory subject in the national school curriculum. However, only two schools (1 public and 1 private) had a written regulation about physical activity (**Table ****3**). Duration of physical education in public schools was longer than in private schools. All schools had sports fields and provided sports equipment for the students. However, some schools (2/6) acknowledged the need of sports field for the students as the schools are surrounded by streets in which many vehicles passing by.

“The play area outside the building or school is very inadequate because it is full of traders selling, even if there are no traders the place is also not suitable as a play area considering that many vehicles pass by”

PE teacher (school 3).

They also encouraged students to participate in sports competition and physical activities. One public school motivated its students to walk or use bicycles to school. All PE teachers asserted that the physical activity policy had been quite effective in their schools, as shown by the attainment of awards in some sports competitions as well as a large number of students who walk from home to school. Five schools rated their teachers as being a very good role model in physical activities through their frequent involvement in the students’ physical activities. One of the private schools held monthly events for teacher–student physical activities.

## Discussion

The PSEA tool can survey many potential obesogenic factors in the primary school environment in a lower-middle income country like Indonesia. The obesogenic factors are reflected in the high availability of the high-calorie, low-nutrient foods; limited choices of healthier foods and higher prices for healthier foods; limited school policies on healthy diet and physical activities; and the lack of understanding by the stakeholders about healthy eating and availability of healthy foods for children at school. We obtained the result from six schools, in which two reflected those in moderate SES area, and the other four in higher SES area. In terms of obesogenic factors, those schools in moderate SES area admitted promoting healthy meal in daily basis, while those in higher level occasionally or never had promotion. Nevertheless, school catering was more accessible in higher SES, compared to moderate SES. We assumed that schools had more control to provide healthy menu for children if they prepared catering.

Availability of food determines its consumption. Access to food was relatively easy for the students in the current study because all schools had a canteen inside. However, food types consistently available in the school environment were mostly rich in calories and sugar. This is similar to the results of a study among schools in Terengganu, Malaysia [17], and Kolkata, India [18]. These findings are also in accordance with the Indonesian Basic Health Research 2018 report [19], which states that among children age > 3 years, 40% consumed high-sugar foods once or more per day, and the highest consumption was among children age 3–14 years. Moreover, approximately 61% also consumed sweet beverages once or more per day, 42% consumed high-fat food once or more per day, and > 96% of children age 5–14 years consumed less than the recommended portions of fruits/vegetables per day. Our study observed that the favorite foods and drinks among students were various kinds of sugar-sweetened beverages in the school canteen as well as deep-fried foods sold by street vendors.

An individual’s food consumption pattern is highly correlated with the individual’s social and economic situation. For example, urbanization has provided opportunities for international supply chains and fast-food chains to operate, displacing traditional markets and local foods [20]. Our observation discovered western fast-food menu like crispy chicken, sausages and spaghetti are among the children’s favorite food at school.

People will consume the foods that are affordable for them, meaning that the food price remains below their income allocated for food. Studies showed that poor families spent between 50%–80% of their income on food whereas middle class families spent 35%–65% of their income [21, 22]. Based on income, people have choices to spend their food budget on healthy or unhealthy foods. Studies from several high-income countries revealed that the price for a healthy diet was higher than that for an unhealthy diet [23, 24]; thus, people with a low income who were less educated preferred to eat an unhealthy diet. However, our study found that the private school canteens, where students are mostly from very high socioeconomic homes, did not provide healthier food compared to those in the public schools.

The average income of Indonesia is about 1.4 million Indonesian rupiah (IDR) (approximately 100 US dollars) monthly [25], and the average expenditure for food consumption is around 0.5 million IDR (approximately 35 US dollars) per month [26], meaning that a household approximately spends 35% of their income for food. A student’s expense for food is highly dependent on the parents’ income. A local study in Central Java found nearly all students received pocket money routinely from their parents, with average expenditure 7915 IDR (± 4288) or around 50–60 cents [27]. The current study did not inquire about the students’ pocket money; however, observations in the school environment showed that the prices of unhealthy foods such as the sweet beverages and fried snacks were generally cheaper than those for the healthier options both in the private and public schools.

Schools hold a major role in promoting healthy eating and physical activities for students. The World Health Organization has already developed the Nutrition-Friendly Schools Initiative for ensuring integrated school-based programs that address the burden of nutrition-related ill health among children. This framework offers opportunities for children to gain access to a healthy environment and promote healthy dietary and physical activity patterns in school, which in return contributes toward improvements in learning and academic achievements [28]. The Ministry of Health of Indonesia has developed technical guidance that emphasizes health education, health services, and healthy environment development integrated into the school curriculum and activities. Some health activities at school environments were endorsed, such as the healthy breakfast program, the eating together program, and “little doctor” program. The Ministry of Education and Culture of Indonesia started a healthy school competition in 1991 to motivate schools to implement healthy lifestyle habits for their students [29]. The present study revealed that schools implemented at least one policy—specifically, the promotion of healthy eating and physical activities as part of the subjects taught in accordance with the national school curriculum. Other than that policy, some schools (2/6) sought to create specific health-promotion activities, yet most were not strictly applied.

The present study asked school principals, PE teachers, students, and canteen staff about their perceptions of healthy nutrition. All interviewees admitted that the schools had limited control of the food choices for their students, that they did not adequately promote healthy diet among students, and that they provided limited choices of healthier foods. Various stakeholders such as parents, community members, non-profit organizations, health professionals, and the private sector should also be involved in improving school health policies [30].

PSEA tool which is an instrument developed and further modified in high-income countries could be used to assess the obesogenicity of the school environment in a lower-middle income country like Indonesia. However, the results must be used with caution because of several limitations. Only one urban city in Indonesia (Jakarta) was evaluated, which may not represent the multicultural demographic in other provinces in Indonesia. Access to food and the types of food may differ among regions because of the culture and availability of food resources. The Indonesian version of PSEA instrument used in this study should be further researched in wider communities, including rural areas, to reflect the broad geographic and cultural variations within Indonesia. Because this study design was qualitative, the number of respondents did not aim to represent the number of schools in the Jakarta area. Furthermore, this study did not gain enough information about the knowledge level of the students, food vendors, and school staff on healthy diet and the reasons why students chose healthy or unhealthy diet.

## Conclusions

PSEA instrument enabled discovery of obesogenic factors in the school environment, such as the easy access to unhealthy food options within schools and its neighborhood and the lack of school policies on the promotion of healthy eating and physical activities to students. Although some support from teachers and parents has been noted, it is still inadequate to motivate students to implement healthy eating and regular physical activities. The Ministry of Education and the Ministry of Health should put forward more coordinated effort to foster the implementation of good nutritional practices and healthy lifestyles at school to improve the health and nutritional status of the students.

## Data Availability

The datasets used and/or analyzed during the current study are available from the corresponding author on reasonable request.

## Abbreviations

PSEA: Primary School Environmental Assessment
PE: Physical education
US: United States
IDR: Indonesian rupiah
SES: Socioeconomic Status
